# Ultrasound classification of thyroid nodules: does size matter?

**DOI:** 10.31744/einstein_journal/2022AO6747

**Published:** 2022-05-06

**Authors:** Raissa Kitaguchi Sakajiri, Antonio Rahal, Miguel José Francisco, Marcos Roberto Gomes de Queiroz, Rodrigo Gobbo Garcia, Leandro Aurélio Liporoni Martins, Domingos Augusto Cherino Malerbi

**Affiliations:** 1 Faculdade Israelita de Ciências da Saúde Albert Einstein Hospital Israelita Albert Einstein São Paulo SP Brazil Faculdade Israelita de Ciências da Saúde Albert Einstein, Hospital Israelita Albert Einstein, São Paulo, SP, Brazil.; 2 Hospital Israelita Albert Einstein São Paulo SP Brazil Hospital Israelita Albert Einstein, São Paulo, SP, Brazil.

**Keywords:** Thyroid gland nodule, Thyroid gland/pathology, ACR-TIRADS, Bethesda, Malignance, Ultrasonography

## Abstract

**Objective:**

To determine whether the size of thyroid nodules in ACR-TIRADS ultrasound categories 3 and 4 is correlated with the Bethesda cytopathology classification.

**Methods:**

Thyroid nodules (566) subclassified as ACR-TIRADS 3 or 4 were divided into three size categories according to American Thyroid Association guidelines. The frequency of different Bethesda categories in each size range within ACR-TIRADS 3 and 4 classifications was analyzed.

**Results:**

Most nodules in both ACR-TIRADS classifications fell in the Bethesda 2 category, regardless of size (90.8% and 68.6%, ACR-TIRADS 3 and 4 respectively). The prevalence of Bethesda 6 nodules in the ACR-TIRADS 4 group was 14 times higher than in the ACR-TIRADS 3 group. There were no significant differences between nodule size and fine needle aspiration biopsy classification in any of the ACR-TIRADS categories.

**Conclusion:**

Size does not appear to be an important criterion for indication of fine needle aspiration biopsy in thyroid nodules with a high suspicion of malignancy on ultrasound examination.

## INTRODUCTION

Thyroid nodules are extremely common in the general population worldwide. The estimated prevalence of nodules detected by palpation is 4% to 7% in women, and 1% in men.^(1)^ However, estimates may be much higher when diagnostic imaging methods are used. Fortunately, in most cases, thyroid nodules are benign. Still, malignancy accounts for 5% to 10% of cases and warrants careful diagnostic investigation. When treated early, thyroid tumors carry an extremely favorable prognosis. Therefore, diagnosis in the early stages of disease has significant impacts on morbidity and mortality.^(2,3)^

Ultrasound examination is the most commonly used tool for investigation of thyroid nodules. Wide use of this imaging modality led to the need for standardized analysis of images potentially associated with higher risk of malignancy. In an effort to create a classification system aimed to facilitate the identification of malignant nodules in ultrasound images, the Korean Thyroid Imaging Reporting and Data System (K-TIRADS, or K-TR) was proposed in 2011.^(4)^ This pioneer classification system is based on four risk stratification criteria: echogenicity, shape, presence of microcalcifications and margins. Based on these parameters, thyroid nodules are scored 1 to 5 in the TIRADS (TR) system. The higher the score, the higher the risk of malignancy and the indication for fine needle aspiration (FNA) biopsy.

Until 2016, the indication of FNA of thyroid nodules was based on the TR classification. In 2017, the American College of Radiology (ACR) published a modified version of this classification system (ACR-TR). In this novel system, nodules are also scored 1 to 5. However, a fifth risk criterion - nodule composition - was included.^(5)^

**Figure 1:**
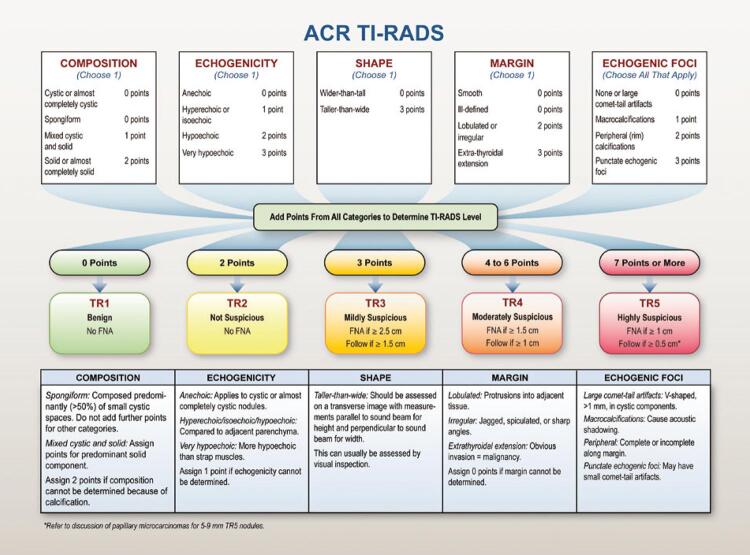
ACR-TR diagnostic matrix for ultrasound classification of thyroid nodules from 2017 White Paper of the ACR TI-RADS Committee

Source: original from Tessler FN, Middleton WD, Grant EG, Hoang JK, Berland LL, Teefey SA, et al. ACR Thyroid Imaging, Reporting and Data System (TI-RADS): white paper of the ACR TI-RADS Committee. J Am Coll Radiol. 2017;14(5):587-95.^(5)^

In the 2015 guidelines, the American Thyroid Association (ATA)^(6)^ proposed the combination of nodule size and ultrasound features for FNA indication. According to these guidelines, ACR-TR 3, 4 and 5 nodules should be stratified according to size (largest diameter, 0.5cm to >2.5cm) for FNA indication (Figure 1).

The predictive power of the ACR-TR system relative to the Bethesda cytopathology classification^(7)^ has not been well established, as shown by heterogeneous findings reported in recent literature.^(8-10)^ However, suspicious imaging findings are thought to be correlated with cytologic criteria of malignancy to a certain degree.

## OBJECTIVE

To determine whether the size of thyroid nodules in ACR-TIRADS ultrasound classification categories 3 and 4 is correlated with the Bethesda cytopathology classification.

## METHODS

This study was based on data from an initial study carried out at *Hospital Israelita Albert Einstein* (HIAE), São Paulo (SP), in 2016. That study compared the K-TR ultrasound classification with cytologic findings (Bethesda reporting system) of one thousand nodules submitted to retrospective and consecutive analysis between 2011 and 2014.^(8)^ Given the methodology adopted in this study (consecutive sampling), the criteria for FNA indication were defined by assistant physicians in each case, and therefore do not reflect the original 2016 publication or any preestablished experimental criteria.

Nodules included in the aforementioned publication were reclassified according to the ACR-TR system. This sample comprised 566 nodules (295 ACR-TR 3 and 271 ACR-TR 4 nodules). Nodules (566) were subclassified by size within each ACR-TR categories, as per ATA guidelines,^(6)^ as follows: ACR-TR 3 nodules, <1.5cm, 1.5 to 2.4cm and ≥2.5cm; ACR-TR 4 nodules, <1cm, 1 to 1.4cm and ≥1.5cm. Subgroups were analyzed according to the Bethesda classification and participant characteristics, such as age and sex.

Data were analyzed using the nonparametric statistical test χ^2^ complemented with the Fisher’s exact test (expected frequency less than 5). The IBM (SPSS), version 20.0, was used and the level of significance set at 5%. The variables sex and age were analyzed using descriptive statistics only.

## RESULTS

The caseload comprised 421 nodules in women (74.4%) and 144 in men (25.4%). In one case, sex was not reported. Age distribution was as follows: 27.4%, 20 to 39 years; 50.9%, 40 to 59 years; 19.8%, 60 to 79 years; 1.9%, 80 years or older.

Data of ACR-TR 3 nodules (295) are shown in table 1: 149 (50.5%) were smaller than 1.5cm, 103 (34.9%) measured 1.5 to 2.4cm and 43 (14.6%) measured 2.5cm or more. Most ACR-TR 3 nodules (90.8%) were classified as Bethesda 2. Only three ACR-TR 3 nodules (1.0%) fell in the Bethesda 6 category. None of ACR-TR 3 nodules was classified as Bethesda 4 or 5. Size range distribution was similar across Bethesda categories. The comparative analysis of size ranges and Bethesda classification of ACR-TR 3 nodules failed to reveal significant differences.

**Table 1:** Nodules ACR-TIRADS 3 in each Bethesda classification

Bethesda	ACR-TIRADS 3		
	<1.5cm n=149 (%)	1.5-2.4cm n=103 (%)	≥2.5cm n=43 (%)
1	4 (2.7)	1 (1.0)	0
2	135 (90.6)	92 (89.3)	41 (95.4)
3	8 (5.4)	10 (9.7)	1 (2.3)
4	0	0	0
5	0	0	0
6	2 (1.3)	0	1 (2.3)
Fisher’s exact test	p=0.347		

Results expressed as n (%).

Characteristics of ACR-TR 4 nodules (271) are shown in table 2: 106 (39.1%) were smaller than 1.0cm, 81 (29.9%) measured 1.0 to 1.4cm, and 84 (31.0%) measured 1.5cm or more. Most ACR-TR 4 nodules (68.6%) were also classified as Bethesda 2. Relative to ACR-TR 3, a much larger number of ACR-TR 4 nodules (39; 14.4%) fell in the Bethesda 6 category. As with the ACR-TR 3 classification, none of ACR-TR 4 nodules fell in the Bethesda 5 category. Nodule size distribution was similar across Bethesda categories 2 and 6. The comparative analysis of size ranges and Bethesda classification of ACR-TR 4 nodules also failed to reveal significant differences.

**Table 2:** Nodules ACR-TIRADS 4 in each Bethesda classification

Bethesda	ACR-TIRADS 4		
	<1cm n=106 (%)	1-1.4cm n=81 (%)	≥1.5cm n=84 (%)
1	8 (7.6)	0	2 (2.4)
2	72 (67.9)	53 (65.4)	61 (72.6)
3	9 (8.5)	13 (16.1)	8 (9.5)
4	3 (2.8)	3 (3.7)	0
5	0	0	0
6	14 (13.2)	12 (14.8)	13 (15.5)
Fisher’s exact test	p=0.087		

Results expressed as n (%).

**Figure 2:**
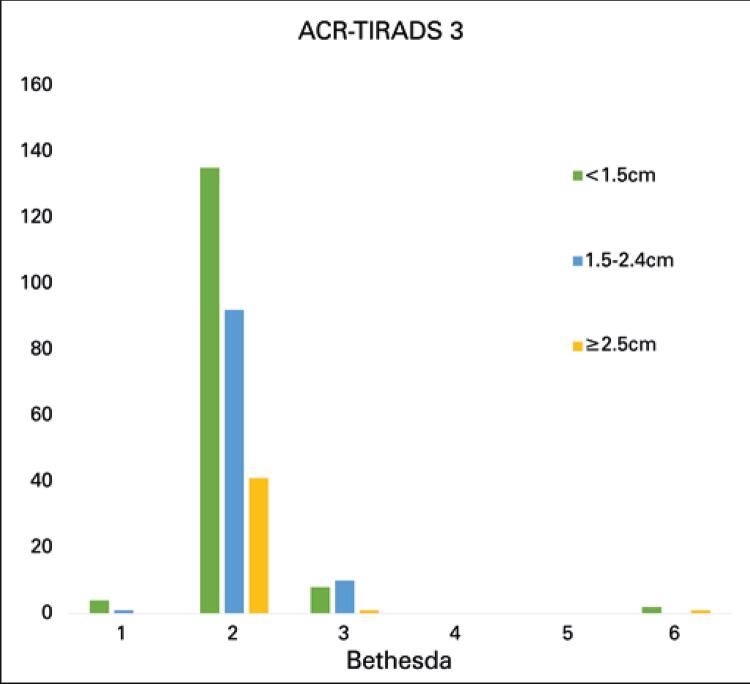
Size distribution of ACR-TR 3 nodules per Bethesda category

**Figure 3:**
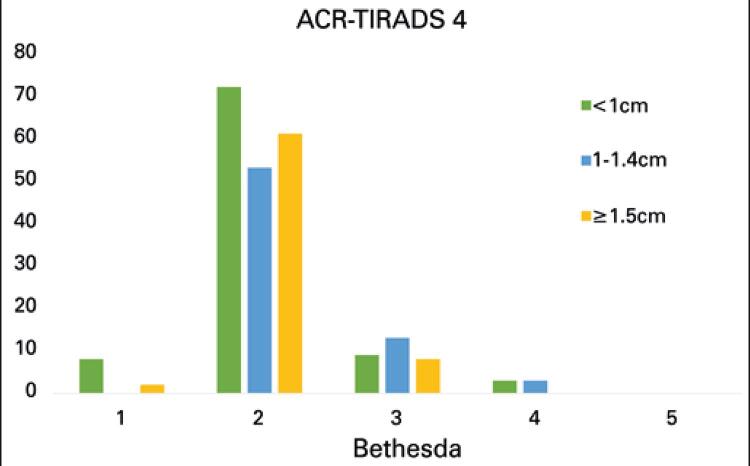
Size distribution of ACR-TR 4 nodules per Bethesda category

Figures 2 and 3 show cytologic findings in ACR-TR classification categories 3 and 4, respectively. These graphic representations provide qualitative support to statistical results.

## DISCUSSION

In several guidelines, thyroid nodule size is used as a parameter for FNA indication.^(6,11,12)^ The ATA proposes the following size cutoffs for FNA indication within ACR-TR classification categories: ACR-TIRADS 3, ≥2.5cm, ACR-TIRADS 4 ≥1.5cm and ACR-TIRADS 5 ≥1.0cm. These indications are based on relations between nodule size and risk of neoplasia reported in different studies. In one of these studies,^(13)^ a nonlinear correlation between risk of malignancy and nodule size was detected in nodules smaller than 2cm, but not in larger nodules. In another, a retrospective case-controlled study^(14)^ with 8,806 patients, nodule size ≥2cm was significantly correlated with risk of neoplasia, among other ultrasound variables. However, in those studies, malignancy was defined according to histologic rather than cytologic criteria. Loss of predictive value of FNA in nodules larger than 3 to 4cm has been reported. In two studies,^(15,16)^ FNA of nodules measuring 4cm or more yielded false negative results in more than 50% of cases. Similar data have been described in a different study with nodules measuring 3cm or more,^(17)^ in which the value of ACR-TR for improved diagnostic accuracy in these cases was emphasized.

Results derived from this caseload confirm the relation between risk of malignancy and ACR-TR classification. The number of Bethesda 6 nodules was 14 times larger in the ACR-TR 4 relative to the ACR-TR 3 group (14.4% and 1%, respectively). However, most nodules (80.2%) fell in the Bethesda 2 category. These findings are consistent with the existing literature reporting that 79.3% to 85.4% of TR 3 or 4 nodules are Bethesda 2,^(8-10)^ and substantiate the general notion that ACR-TR is correlated with risk of malignancy in FNA.^(9,18)^ The ACR-TR system is thought to have high sensitivity and low specificity and can therefore be used as a screening tool. In the original caseload with one thousand nodules published in 2016,^(8)^ Bethesda 6 nodules accounted for 0.8%, 1.7%, 13.4, 68.2% and 91.3% of nodules in K-TR categories 2, 3, 4A to 4C and 5, respectively. More recent data revealed Bethesda 6 rates of 0%, 0%, 2.2% and 21.5% in ACR-TR categories 2, 3, 4 and 5, respectively.^(9)^

Nodule size was not correlated with risk of malignancy. In this study, most ACR-TR 3 nodules fell in the Bethesda 2 category, regardless of size (90.6% smaller than 1.5cm, 89.3% between 1.5 and 2.4cm, and 95.4% larger than 2.5cm). The fact that, out of three Bethesda 6 nodules, two were smaller than 1.5cm and one larger than 2.5cm, demonstrates that smaller nodules are not necessarily less malignant.

Similar behavior was observed in the ACR-TR 4 group, with relatively even size distribution across Bethesda 2 (67.9% smaller than 1cm, 65.4% between 1 and 1.4cm and 72.6% larger than 1.5cm) and Bethesda 6 (13.2% smaller than 1cm, 14.8% between 1 and 1.4cm and 15.5 larger than 1.5cm) categories.

In both ACR-TR classifications considered, statistical analysis confirmed the lack of significant differences among the three size ranges within each Bethesda category.

Findings of this study are in keeping with those of a recent publication.^(9)^ in which 2,306 nodules classified as ACR-TR 3, 4 or 5 were stratified according to two size ranges, above and below the cutoff for FNA indication as per 2015 ATA guidelines. Nodule size was not significantly associated with the risk of cytologic malignancy in any of the three ACR-TR classification categories. However, results of that study may have been biased: given smaller nodules are often not submitted to FNA, the risk of malignancy in these nodules may be lower than the risk reported.

Similar conclusions from other studies^(19,20)^ investigating correlations between nodule size and histologic diagnosis of malignancy support the notion that nodule size should not be accounted for in the indication of FNA.

The low prevalence of nodules with FNA Bethesda 4 or 5 decreased the power of statistical tests and is a limitation of this study. Also, data in this sample were collected in a reference service and may lack representativeness.

## CONCLUSION

In this service, the size of thyroid nodules was not correlated with the Bethesda cytopathology classification. Use of nodule size as a criterion for indication of fine needle aspiration biopsy does not add benefit to ultrasound criteria, including the ACR-TIRADS classification.
